# Degree of Uncertainty in Reporting Imaging Findings for Necrotizing Enterocolitis: A Secondary Analysis from a Pilot Randomized Diagnostic Trial

**DOI:** 10.3390/healthcare12050511

**Published:** 2024-02-21

**Authors:** Alain Cuna, Disa Rathore, Kira Bourret, Erin Opfer, Sherwin Chan

**Affiliations:** 1 Division of Neonatology, Children’s Mercy Kansas City, Kansas City, MO 64108, USA; 2 School of Medicine, University of Missouri-Kansas City, Kansas City, MO 64108, USA; 3 School of Medicine, Kansas City University, Kansas City, MO 64106, USA; 4 Department of Radiology, Children’s Mercy Kansas City, Kansas City, MO 64108, USA

**Keywords:** necrotizing enterocolitis, radiography, ultrasound, radiology report, language, diagnostic certainty, uncertainty

## Abstract

Diagnosis of necrotizing enterocolitis (NEC) relies heavily on imaging, but uncertainty in the language used in imaging reports can result in ambiguity, miscommunication, and potential diagnostic errors. To determine the degree of uncertainty in reporting imaging findings for NEC, we conducted a secondary analysis of the data from a previously completed pilot diagnostic randomized controlled trial (2019–2020). The study population comprised sixteen preterm infants with suspected NEC randomized to abdominal radiographs (AXRs) or AXR + bowel ultrasound (BUS). The level of uncertainty was determined using a four-point Likert scale. Overall, we reviewed radiology reports of 113 AXR and 24 BUS from sixteen preterm infants with NEC concern. The BUS reports showed less uncertainty for reporting pneumatosis, portal venous gas, and free air compared to AXR reports (*pneumatosis*: 1 [1–1.75) vs. 3 [2–3], *p* < 0.0001; *portal venous gas*: 1 [1–1] vs. 1 [1–1], *p* = 0.02; *free air*: 1 [1–1] vs. 2 [1–3], *p* < 0.0001). In conclusion, we found that BUS reports have a lower degree of uncertainty in reporting imaging findings of NEC compared to AXR reports. Whether the lower degree of uncertainty of BUS reports positively impacts clinical decision making in infants with possible NEC remains unknown.

## 1. Introduction

Necrotizing enterocolitis (NEC) is an acute and life-threatening disease characterized by uncontrolled inflammation of the premature intestinal tract [1]. Despite extensive research, NEC remains a leading cause of morbidity and mortality in preterm infants. About 10% of preterm infants with birth weight less than 1500 g are expected to develop NEC, while mortality from NEC can be as high as 20% to 45%. Infants who survive NEC are also at high risk for serious morbidities including prolonged hospital stay, short bowel syndrome, poor nutrition and growth, and poor neurodevelopmental outcomes [2].

Because clinical and laboratory features are non-specific, the diagnosis of NEC is challenging and is typically dependent on identifying pathognomonic signs on imaging [3,4,5]. The presence of pneumatosis intestinalis or portal venous gas is generally sufficient to make a diagnosis in infants with clinical suspicion of NEC, while the presence of free air typically indicates NEC complicated by intestinal perforation that requires surgical intervention. Traditionally, abdominal radiographs (AXRs) have been used as the standard imaging test to identify the pathognomonic imaging findings of NEC. But, in recent years, bowel ultrasound (BUS) has steadily emerged as a helpful adjunct to AXR to aid in NEC diagnosis [6,7,8]. As a non-invasive imaging modality, BUS is free from radiation and well tolerated by preterm infants [9]. Like AXR, BUS can identify pneumatosis, portal venous gas, and free air. In addition, BUS allows for real-time assessment of the intestinal wall, vascular perfusion, peristalsis, and abdominal fluid [10].

Unlike diagnostic tests that have discrete empiric values (such as complete blood counts or chemistry panel), imaging tests require interpretation by the radiologist, the findings of which are then conveyed to the ordering clinician via a written imaging report. The interpretation of imaging tests is not binary [11]. Often, there exists a degree of uncertainty regarding whether imaging findings are normal or abnormal [12]. As such, the language used in imaging reports needs to accurately convey the radiologist’s uncertainty in identifying the presence or absence of important imaging findings [13]. Many factors can influence the degree of uncertainty conveyed in imaging reports, including technical limitations, insufficient clinical data, lack of established standards, education and training variability, and fear of malpractice [14]. Using language with a high degree of uncertainty can be a common source of miscommunication and misunderstanding that can lead to diagnostic errors, delayed clinical decision making, and adverse outcomes [15,16,17,18].

The degree to which uncertain language is used in reporting imaging findings for NEC can have important implications for clinical care. For example, highly uncertain phrases such as “cannot rule out pneumatosis” could influence the neonatologist’s plan for treatment even when overall clinical suspicion for NEC is low. Conversely, decreasing uncertainty helps provide clarity, improve communication, and convey the diagnostic confidence of the radiologist regarding the presence or absence of key imaging findings. To the best of our knowledge, the degree to which uncertainty complicates diagnostic imaging reports for NEC has not been characterized.

In this study, we sought to quantify the degree of uncertainty in imaging reports derived from a pilot diagnostic randomized clinical trial (RCT) of infants with suspected NEC who were evaluated with either AXR alone or AXR + add-on BUS [19]. We hypothesized that BUS reports have lower uncertainty than AXR reports and that uncertainty in AXR reports could be lowered by adding information from BUS. We also evaluated whether uncertainty in imaging reports is affected by the experience of the interpreting radiologist.

## 2. Materials and Methods

### 2.1. Study Design

This was a secondary analysis using data from a previously completed pilot diagnostic RCT [19]. The study population comprised preterm infants ≤ 32 weeks’ gestation at birth with suspected NEC who were randomized to either standard imaging with AXR alone or experimental imaging with AXR + add-on BUS. Infants with abdominal wall defects that prohibit 4-quadrant evaluation using BUS were excluded from the study. We evaluated uncertainty by analyzing de-identified imaging reports of AXR and BUS studies acquired during this pilot RCT. The institutional review board at our local institution approved the secondary analysis of this pilot RCT with waiver of informed consent.

### 2.2. Study Setting

The original pilot RCT was conducted in a level IV neonatal intensive care unit of a tertiary free-standing children’s hospital with 24/7 coverage by neonatologists, pediatric radiologists, and pediatric surgeons. Infants randomized to the AXR group were evaluated with a portable AXR as per standard of care and consisted of anteroposterior view, with additional cross-table or left lateral decubitus view per neonatologist discretion. Infants randomized to the AXR + BUS group had a BUS performed within 6 h of the standard of care AXR. The BUS protocol consisted of standard grayscale, color Doppler, and spectral Doppler images of the abdomen supplemented with cine acquisitions in both transverse and sagittal planes. All imaging tests were performed by radiology technologists and interpreted by pediatric radiologists who were board-certified in pediatric radiology. At the time of the pilot RCT, the use of standard reporting templates (Table 1) for reporting NEC findings was encouraged but not mandated. These templates could be edited by the radiologist to reflect their interpretation regarding the presence or absence of a finding as well as their degree of certainty.

### 2.3. Determination of Uncertainty

Three investigators (AC, DR, and KB) independently reviewed de-identified imaging reports and assigned uncertainty scores using a 4-point Likert scale adopted from the study by Reiner [20]. In this scale, a score of 1 denotes the lowest level of uncertainty; a score of 2 denotes minimal level of uncertainty; a score of 3 denotes intermediate level of uncertainty; and a score of 4 denotes the highest level of uncertainty (Table 2). We limited our evaluation to pneumatosis, portal venous gas, and free air because these findings are most specific for NEC and because these findings are assessed in both AXR and BUS. Disagreements in scoring were discussed as a group and resolved by consensus.

### 2.4. Outcomes and Variables of Interest

Our primary outcome was uncertainty scores for pneumatosis, portal venous gas, and free air. Secondary outcomes included proportion of imaging reports with all three imaging findings present and proportion of imaging reports that used standardized reporting templates. We also assessed whether other factors such as the addition of BUS and differences in radiology personnel (involvement of trainees, years of experience and subspecialty of the interpreting radiologist) impacted uncertainty scores.

### 2.5. Statistical Analysis

Data are presented as mean and standard deviation, median and interquartile range, or numbers and percentages. Normality of data was determined using the Shapiro–Wilk test. Differences in primary and secondary outcomes between the imaging modality (AXR vs. BUS) and randomization arm (AXR vs. AXR + BUS) were evaluated using the Mann–Whitney U test and chi-square test, as appropriate. To determine the effect of add-on BUS on quality of imaging reporting, uncertainty scoring of infants with AXR reports completed within 6–8 h before and after add-on BUS were compared using a paired Wilcoxon signed-rank test. To determine the effect of trainee involvement as well as years of experience and subspecialty of the interpreting radiologist on the uncertainty of medical reporting, uncertainty scores were first dichotomized to low uncertainty (scores of 1 or 2) or high uncertainty (scores of 3 or 4) followed by binomial logistic regression. All analyses were performed in SPSS software (IBM SPSS Statistics for Windows, Version 24.0, Armonk, NY, USA), and statistical significance was set at *p* < 0.05.

## 3. Results

Overall, sixteen preterm infants with concern for NEC were randomized as part of the original pilot RCT. The mean gestational age and mean birth weight for the entire cohort was 27.2 ± 2.2 weeks and 1020 ± 373 g, respectively. The baseline characteristics were comparable between the groups (Table 3). The eight infants randomized to the AXR arm had 49 AXRs, while the eight infants randomized to the AXR + BUS arm had 64 AXRs and 24 BUSs. Thus, overall, we reviewed 113 AXR reports and 24 BUS reports. We had six instances of disagreement in uncertainty scoring—four with AXR reports and two with BUS reports.

**Figure 1:**
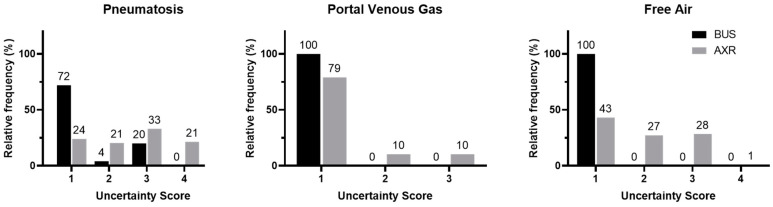
Frequency distribution of uncertainty scores for reporting pneumatosis, portal venous gas, and free air comparing BUS versus AXR. In all three imaging findings of NEC, BUS (black bars) was observed to have lower uncertainty scores than AXR (gray bars).

The uncertainty scores for each main NEC finding described in the BUS and AXR reports are displayed in Figure 1. Overall, BUS had lower uncertainty scores for reporting each of the three main NEC findings than AXR (*pneumatosis*: 1 [1–1.75) vs. 3 [2–3], *p* < 0.0001; *portal venous gas*: 1 [1–1] vs. 1 [1–1], *p* = 0.02; *free air*: 1 [1–1] vs. 2 [1–3], *p* < 0.0001).

The BUS reports described all three main NEC imaging findings (pneumatosis, portal venous gas, and free air) in 96% of the reports (Table 4). The only exception was the omission of reporting about free air on one BUS report. In contrast, the AXR reports described all three main NEC findings in only 52% of the reports. The NEC finding most frequently absent in the AXR reports was portal venous gas (Table 4). The BUS reports also used a standardized reporting template more frequently than the AXR reports (Table 4).

To determine whether the addition of BUS impacted AXR reporting, we first compared the AXR reports from infants in the AXR arm to infants in the AXR + BUS arm. We found that the completeness of describing the three main NEC findings was similar between the two arms (Table 5). The use of a standardized template for NEC impression was also similar (Table 5).

**Figure 2:**
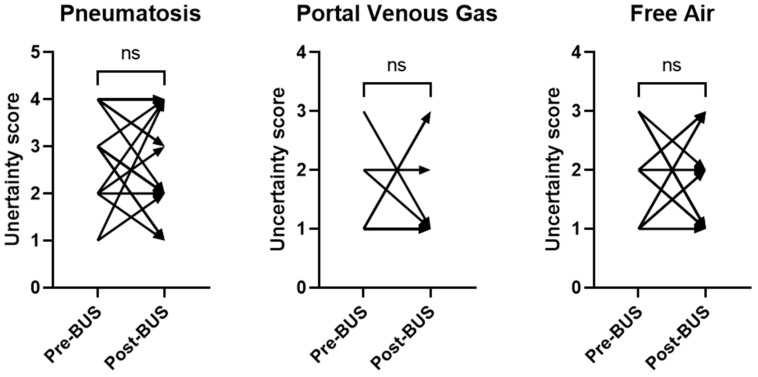
Change in uncertainty scores in AXR reports before and after add-on BUS. For all three imaging findings, uncertainty scores of AXR reports did not change significantly between baseline (pre-BUS) and after paired BUS examination (post-BUS). ns = not significant.

We then evaluated AXRs that had a paired BUS (within 6 h of AXR) in the AXR + BUS arm. The change in uncertainty score from the AXR reports for pneumatosis, portal venous gas, and free air pre- and post-BUS is displayed in Figure 2. A paired Wilcoxon signed-rank test revealed no significant change in uncertainty scoring from AXRs conducted before and after the BUS study (Figure 2).

Lastly, we evaluated whether differences in experience, specialization, and trainee involvement played a role in the lower uncertainty scoring of BUS compared to AXR. Overall, 22 pediatric radiologists interpreted all imaging tests in the study. Of these, 11 reported on only AXR, 1 reported on only BUS, and 10 reported on both AXR and BUS. Their experience ranged from 5 to 24 years, and their specialization included body, cardiac, brain, interventional radiology, fetal, and musculoskeletal. Eight reports had trainee involvement. Using binomial logistic regression, we found that only BUS was associated with increased likelihood of lower-uncertainty reports (OR 5.64, 95% CI 1.88–16.9). Years of experience, specialization, and trainee involvement did not influence uncertainty scores.

## 4. Discussion

In this study, we sought to determine the level of uncertainty in radiology reports of preterm infants who underwent diagnostic imaging for suspected NEC. We found that the BUS reports had significantly less uncertainty in reporting pneumatosis, portal venous gas, and free air compared to the AXR reports. The rate of completeness in reporting imaging findings of NEC and the rate of using standardized reporting templates were also significantly higher in the BUS reports than in the AXR reports. The addition of paired BUS did not decrease the high level of uncertainty in the AXR reports. Years of experience, radiology subspecialty, or trainee involvement also did not affect the level of uncertainty.

The lower level of uncertainty in BUS reports may be explained by the superior technical images of BUS over AXR in NEC evaluation. Because ultrasound waves cannot pass through air, pneumatosis intestinalis and portal venous gas are easily detected as bright, echogenic foci on BUS. Several studies have found that BUS can identify portal venous gas and pneumatosis intestinalis earlier than AXR [21,22,23]. BUS is also capable of the real-time assessment of bowel loops in cross-section, allowing an opportunity to evaluate bowel wall thickness, peristalsis, and perfusion (with Doppler interrogation) [24]. In addition, BUS assessment occurs over ~30 min, whereas AXR is a one-time assessment [9,25]. Therefore, BUS is better able to capture dynamic and intermittent processes like portal venous gas. A constellation of thinning of the bowel wall, decreased or absent peristalsis, and decreased or absent perfusion can be indicative of impending bowel perforation [10,26]. The advantage of BUS in assessing abdominal structures in more detail compared to AXR is likely a major driver of the lower level of uncertainty we found in BUS reports.

In a similar fashion, the high level of uncertainty in AXR reports can be explained by the inherent challenges in identifying findings of NEC on plain film radiography with consistency. Sharma et al. [27] used computer-aided simulation to assess radiology trainees and found that only 28% were able to correctly identify pneumatosis intestinalis on standardized test AXRs. Di Napoli et al. [18] found poor reliability for NEC diagnosis and individual NEC imaging findings even among three expert radiologists who independently reviewed 297 AXRs of infants with and without NEC. Other studies have demonstrated similar poor agreement in identifying pathognomonic signs of NEC on AXR among radiologists, neonatologists, and pediatric surgeons [15,16,17]. These studies suggest that the inherent limitations of radiography play a major role in the higher level of uncertainty found in AXR reports for NEC evaluation.

A secondary objective of our study was to investigate whether the addition of BUS would help decrease the level of uncertainty in AXR reports. Contrary to our hypothesis, we found that the uncertainty in the AXR reports remained unchanged despite the addition of BUS. One possible explanation for our results is that the AXRs were interpreted independently and without consideration of the preceding BUS results. Although we cannot exclude this possibility, we believe this is unlikely because it is standard practice to review prior images for accurate interpretation of any follow-up imaging for NEC. Instead, a more likely explanation is that the inherent limitations of AXR preclude radiologists from using more certain language for reporting NEC findings. Other investigators have used standardized reporting tools to help overcome the challenges and limitations of AXR in NEC imaging. One such tool is the Duke Abdominal Assessment Scale (DAAS), a standardized 10-point numerical scale for reporting abnormal radiographic findings in NEC [28]. Initial studies demonstrated substantial improvement in inter-observer and intra-observer agreement among radiologists following DAAS implementation [29]. However, other studies found that a fair degree of disagreement persisted even with DAAS implementation [30]. Taken together, these findings suggest that strategies such as standardized reporting tools and adjunct BUS may not be sufficient to overcome the inherent limitations of AXR that result in the high level of uncertainty of reporting NEC findings.

We also investigated whether the experience of the interpreting radiologist impacted the level of uncertainty in imaging reports. In a single-center study, Callen et al. [31] used natural language processing to characterize the use of uncertainty terms of over 640,000 radiology reports by 171 interpreting radiologists. While substantial variability in the use of uncertainty terms was observed, the degree of variability could not be explained by differences in years of experience of radiologists. Similar results were observed by Crombe et al. [32] in a multicenter study of over 30,000 computed topography scans and magnetic resonance imaging interpreted by 165 radiologists. Despite having a smaller sample size, our study mirrors the findings of these studies in demonstrating no correlation between the use of uncertain language on imaging reports and the experience of the interpreting radiologist.

One way to potentially decrease the uncertainty in imaging reports is the addition of diagnostic certainty scales that convey the radiologist’s confidence in their interpretation [33]. Several studies have reported how the voluntary adoption of diagnostic certainty scales by radiologists resulted in a modest to moderate increase in its use, suggesting the feasibility of this practice [34,35,36]. More importantly, studies have found that the radiologist’s diagnostic certainty score correlated strongly with the subsequent confirmation of disease. In one study, Godwin et al. [37] employed diagnostic certainty scoring for the presence or absence of appendicitis based on CT scan findings and found that diagnostic certainty scoring correlated strongly with post-operative pathology. In another study, Wibmer et al. [38] demonstrated how the radiologist’s diagnostic certainty score for the routine staging MRI of prostate cancer correlated with subsequent histopathology for extracapsular extension. Taken together, these studies indicate that the adoption of diagnostic certainty scales in imaging reports is feasible and can potentially increase the accuracy of diagnosis.

Furnishing more clinical information to the ordering physician can also decrease uncertainty by improving communication and offering valuable context to radiologists. In a retrospective study, Maizlin and Somers [39] investigated the impact of additional information from radiology technician notes compared to the original information from the imaging order requisition. Of the 250 radiographs and ultrasound tests reviewed, 47% of imaging order requisitions were either incomplete or absent. The added information from technician notes was deemed by radiologists to provide important value in more than two-thirds (173/250 = 69%) of the reviewed cases. In another study, Lacson et al. [40] compared the clinical information on imaging order requisition with the clinical information on the provider notes. Of the 315 magnetic resonance imaging and computed tomography scans reviewed, information on order requisitions was incomplete in 81% and discordant in 42% of reviewed cases. Incomplete and discordant clinical information was deemed by reviewing radiologists to negatively impact interpretation in 43% (135/315) of cases. Several other studies [41,42], including a systematic review [43], showed similar findings that the improved availability of clinical information is beneficial for optimal interpretation.

Whether improving uncertainty in reporting NEC imaging findings has important implications in clinical care remains unknown. The results from the original pilot RCT showed no difference in clinical outcomes between infants randomized to AXR versus AXR + BUS imaging [19]. However, this pilot study was designed for feasibility and not powered to detect differences in clinical outcomes. Studies of ultrasound for pediatric appendicitis suggested that uncertain interpretation was associated with poorer outcomes and higher medical costs compared to situations when more certain interpretation was provided [44,45]. Given the similarities between appendicitis and NEC, it is plausible that increasing certainty in reporting for NEC could translate to improved patient outcomes, particularly reductions in antibiotic use, bowel rest, and intravenous nutrition.

Our study has several strengths. To the best of our knowledge, this is the first study that measured the degree of uncertainty in the language used in reports of diagnostic imaging tests for NEC. Our results could be useful as reference data for future research or quality improvement studies regarding the uncertainty in imaging reports for NEC. Our results also provide supporting evidence that higher diagnostic certainty could be another advantage of BUS over AXR for evaluating NEC. The secondary use of data from a randomized diagnostic trial increases the validity of our results, as randomization eliminates the possibility that the lower uncertainty scores in the BUS reports could have been due to selection bias. Another strength of the study is our analysis of other factors that could influence uncertainty scores, such as the addition of paired BUS and the experience of the interpreting radiologist.

Our study is limited by the small sample size and the single-center study design of the original randomized diagnostic trial, which experienced challenges in recruitment due to the COVID-19 pandemic [19]. Despite this limitation, our study evaluated 137 imaging reports, which provided sufficient power to detect a significant difference in our primary outcome. Another limitation is that our study was conducted in a tertiary children’s hospital with pediatric radiologists who were adept at interpreting AXR and BUS studies for NEC. Whether similar uncertainty scores are seen in institutions that lack pediatric radiologists is unknown. A third limitation is that we cannot exclude the possibility that the wide variation in uncertainty could be related to the differential use of structured reporting templates between BUS and AXR. While reporting templates are available for both AXR and BUS, radiologists may have relied more on standardized reporting templates for BUS because it is a relatively newer modality compared with AXR. Lastly, our study could not determine whether less uncertainty in reporting from BUS leads to improved patient outcomes. Future studies are needed to properly evaluate the relationships among communication of diagnostic uncertainty, management, and improved patient outcomes. 

## 5. Conclusions

In conclusion, we found that BUS reports convey imaging findings of NEC with less uncertainty than AXR reports. These findings suggest that radiologists are more confident in identifying the presence or absence of key NEC findings based on BUS than on AXR. Future studies are needed to determine whether the lower degree of uncertainty with BUS translates to improved care of infants with suspected NEC.

## Figures and Tables

**Table 1:** Reporting templates for NEC evaluation. The AXR template reports on four findings, while the BUS template reports on ten findings.

Imaging Modality	Template
AXR	There are no findings to suggest bowel obstruction, free intraperitoneal gas, or pneumatosis.
	There is no portal venous gas.
BUS	There is no bowel wall thickening (>2.7 mm).
	There is no bowel wall thinning (<1.0 mm).
	There is no bowel wall hyperechogenicity.
	There is no pneumatosis intestinalis.
	There is no portal venous gas.
	There is no pneumoperitoneum.
	There are no focal fluid collections with complex echoes.
	There is no free fluid.
	There is normal bowel wall perfusion.
	Peristalsis is present.

AXR, abdominal radiograph; BUS, bowel ultrasound; NEC, necrotizing enterocolitis.

**Table 2:** Scoring system for determining degree of uncertainty.

Score	Degree of Uncertainty	Example
1	Lowest level of uncertainty. No equivocation.	No pneumatosis.
		No portal venous gas.
		No pneumoperitoneum.
2	Low level of uncertainty. Minimal equivocation.	No definite pneumatosis.
		No supine evidence of pneumoperitoneum.
		No obvious free air.
3	Intermediate level of uncertainty. Intermediate degree of equivocation.	No findings to suggest pneumatosis, portal venous gas, or free air.
		Small area of possible linear lucency along the bowel wall.
		Suggestion of interim mural air within the bowel wall.
4	Highest level of uncertainty. High degree of equivocation.	Cannot exclude pneumatosis.
		No obvious pneumatosis although evaluation is limited.
		Mild mottled lucencies which may represent pneumatosis or stool.

**Table 3:** Characteristics of the study participants from the original pilot trial.

Baseline Characteristics	AXR Group (*n* = 8)	AXR Plus BUS Group (*n* = 8)
Gestational age, weeks	26.9 ± 2.5	27.4 ± 2.1
Birth weight, grams	1056 ± 399	1022 ± 381
Male sex, no. (%)	5 (63)	2 (25)
White race, no. (%)	4 (50)	6 (75)
Small for gestational age, no. (%)	0 (0)	1 (13)
Maternal age, years ^a^	25 ± 7	31 ± 7
Caesarian delivery, no. (%)	3 (38)	5 (63)
Apgar score < 5 at 1 min, no. (%) ^b^	4 (50)	7 (88)
Apgar score < 5 at 5 min, no. (%) ^b^	3 (38)	2 (25)
Antenatal corticosteroids, no. (%)	8 (100)	7 (88)
Surfactant, no. (%)	8 (100)	6 (75)
No. of NEC concern episodes	1 (1–3)	1 (1–3)
No. of imaging studies		
AXR	49	64
BUS	0	24

Data presented as number (percentage), mean ± standard deviation, or median (interquartile range). ^a^ One mother with unknown age; ^b^ two infants with unknown Apgar scores. AXR, abdominal radiograph; BUS, bowel ultrasound; NEC, necrotizing enterocolitis.

**Table 4:** Comparison of reporting metrics between BUS and AXR.

	BUS *n* = 24	AXR *n* = 113	*p*
Complete report, *n* (%)	23 (96)	59 (52)	0.0001
Pneumatosis	24 (100)	111 (98)	1.0
Portal venous gas	24 (100)	67 (59)	0.0001
Free air	23 (96)	95 (84)	0.19
Use of standardized template, *n* (%)	21 (88)	18 (16)	0.0001

Data presented as number (percent); *p* value represents comparison with chi-square test. AXR, abdominal radiograph; BUS, bowel ultrasound.

**Table 5:** Comparison of reporting metrics of AXR reports from infants with and without add-on BUS.

	AXR + BUS Arm *n* = 64	AXR Arm *n* = 49	*p*
Complete report, *n* (%)	35 (55)	24 (49)	0.55
Pneumatosis	63 (98)	48 (98)	1.0
Portal venous gas	42 (66)	25 (51)	0.12
Free Air	54 (84)	41 (84)	0.92
Use of standardized template, *n* (%)	9 (14)	9 (18)	0.54

Data presented as number (percent). *p* value represents comparison with chi-square test. AXR, abdominal radiograph; BUS, bowel ultrasound.

## Data Availability

The data presented in this study are available on request from the corresponding author.
